# Mollaret-like Cells in Patients with West Nile Virus Infection

**DOI:** 10.3201/eid1004.030783

**Published:** 2004-04

**Authors:** Gary W. Procop, Belinda Yen-Lieberman, Richard A. Prayson, Steve M. Gordon

**Affiliations:** * The Cleveland Clinic Foundation, Cleveland, Ohio

**To the Editor:** We have read with interest many of the articles concerning West Nile virus (WNV) published in the July 2003 issue of Emerging Infectious Diseases. Last summer Ohio was one of the leading states with WNV infection in humans. Consequently, requests for tests for this pathogen have increased. Unfortunately, the turnaround time for testing these specimens may be delayed because of shipping difficulties secondary to associates, the limited number of laboratories that can perform these assays, and an increase in requests at testing facilities.

Cytologic examination of cerebrospinal fluid (CSF) from patients with WNV has not been studied. Although cytologic examination of CSF from patients with encephalitis is likely nonspecific, it may provide supportive information of the suspected disease process, and is useful for excluding other conditions, such as neoplasia. Of the 22 patients that were hospitalized at our institution last year with WNV meningoencephalitis, documented by serologic tests and/or reverse transcription-polymerase chain reaction, CSF of 4 of these patients was submitted for cytologic examination. Of these 4, 3 had a sufficient number of cells in the CSF specimen (47, 213, and 495 cell/μL) to afford cytologic examination, whereas one had a paucicellular CSF, with only 2 white blood cells/μl. The cytologic features from the 3 patients >10 cells/μL consistently demonstrated a mixture of lymphocytes at various stages of activation and occasional large monocytic-like cells with cerebriform nuclei reminiscent of the Mollaret cells described in CSF of patients with recurrent meningitis (Figure) (*1*).

**Figure:**
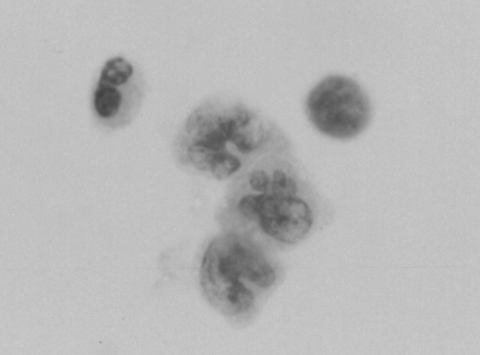
Three Mollaret-like cells are present (center), with a neutrophil (upper left) and a lymphocyte (upper right) in cerebrospinal fluid from a patient with West Nile Virus encephalitis, confirmed by reverse transcription–polymerase chain reaction and serologic testing (Papanicolaou stain; magnification x 500).

Mollaret described cells with enlarged nuclei and cerebreform nuclear contours in CSF of patients with recurrent, aseptic meningitis (*1*). Although he believed these were of endothelial origin, immunohistochemical studies have subsequently shown that they are monocytes (*2*). This type of meningitis, now commonly known as Mollarets meningitis, has been associated with herpes simplex virus encephalitis, but the definitive cause of all cases remains unclear (*3*).

One of the patients infected with WNV meningoencephalitis who had Mollaret-like cells in CSF died. Postmortem neuropathologic examination showed an extensive perivascular lymphocytic infiltrate which contained mononuclear cells consistent with the Mollaret-like cells in CSF. These mononuclear cells were stained with an immunohistochemical stain directed against the CD68 antigen, which supports a monocytic origin (*4*). Further studies are needed to delineate the consistency of Mollaret-like cells in CSF of patients with WNV meningoencephalitis. Finding Mollaret-like cells admixed with activated lymphocytes may be a useful, readily-available test that provides supportive evidence of viral encephalitis in the appropriate clinical setting, until more definitive tests are available.
